# Glycomics expression analysis of sulfated glycosaminoglycans of human colorectal cancer tissues and non-neoplastic mucosa by electrospray ionization mass spectrometry

**DOI:** 10.1590/S1679-45082015AO3477

**Published:** 2015

**Authors:** Ana Paula Cleto Marolla, Jaques Waisberg, Gabriela Tognini Saba, Daniel Reis Waisberg, Fernando Beani Margeotto, Maria Aparecida da Silva Pinhal

**Affiliations:** 1 Universidade Federal de São Paulo, São Paulo, SP, Brazil.; 2 Hospital do Servidor Público Estadual, São Paulo, SP, Brazil.; 3 Faculdade de Medicina do ABC, Santo André, SP, Brazil.; 4 Faculdade de Medicina da Universidade de São Paulo, São Paulo, SP, Brazil.

**Keywords:** Glycosaminoglycans, Proteoglycans, Chondroitin, Dermatan sulfate, Heparitin sulfate, Extracellular matrix, Colorectal neoplasms, Spectrometry, mass, electrospray ionization

## Abstract

**Objective:**

To determine the presence of glycosaminoglycans in the extracellular matrix of connective tissue from neoplastic and non-neoplastic colorectal tissues, since it has a central role in tumor development and progression.

**Methods:**

Tissue samples from neoplastic and non-neoplastic colorectal tissues were obtained from 64 operated patients who had colorectal carcinoma with no distant metastases. Expressions of heparan sulphate, chondroitin sulphate, dermatan sulphate and their fragments were analyzed by electrospray ionization mass spectrometry, with the technique for extraction and quantification of glycosaminoglycans after proteolysis and electrophoresis. The statistical analysis included mean, standard deviation, and Student’s *t* test.

**Results:**

The glycosaminoglycans extracted from colorectal tissue showed three electrophoretic bands in agarose gel. Electrospray ionization mass spectrometry showed characteristic disaccharide fragments from glycosaminoglycans, indicating their structural characterization in the tissues analyzed. Some peaks in the electrospray ionization mass spectrometry were not characterized as fragments of sugars, indicating the presence of fragments of the protein structure of proteoglycans generated during the glycosaminoglycan purification. The average amount of chondroitin and dermatan increased in the neoplastic tissue compared to normal tissue (p=0.01). On the other hand, the average amount of heparan decreased in the neoplastic tissue compared to normal tissue (p= 0.03).

**Conclusion:**

The method allowed the determination of the glycosaminoglycans structural profile in colorectal tissue from neoplastic and non-neoplastic colorectal tissue. Neoplastic tissues showed greater amounts of chondroitin sulphate and dermatan sulphate compared to non-neoplastic tissues, while heparan sulphate was decreased in neoplastic tissues.

## INTRODUCTION

Homeostasis of extracellular matrix depends on the integrity and assembly of collagens, glycoproteins, proteoglycans (PG), and sulfated glycosaminoglycans (S-GAG).^(1-3)^ S-GAG bind to specific proteins altering their essential roles in development, organogenesis, cell growth, cell adhesion, inflammation, angiogenesis modulation, apoptosis, cell cycle control, and growth factor signaling.^(4-7)^Also, S-GAG play vital roles in every step of tumor progression, allowing cancer cells to proliferate, escape from immune response, invade neighboring tissues, and metastasize to distal sites away from the primary site.^(5,8,9)^ Chondroitin sulfate (CS) and dermatan sulfate (DS), both galactosaminoglycans, and heparan sulfate (HS), a glycosaminoglycan, are S-GAG involved in these biological events.^(10-12)^

Several techniques were revised to identify glycans selectively at the reducing end, with a group that could enhance the sensitivity to detect and facilitate chromatographic separations.^(3,13,14)^However, the analysis of S-GAG and PG derived from small amounts of tissue or cells, and the development of methods to characterize these carbohydrates have progressed comparatively slowly.^(1-3)^

Mass spectrometry is an analytical technique used to identify unknown compounds, quantify known materials, and elucidate the chemical and structural properties of molecules, based on the generation of ions that are subsequently detected.^(3,15)^This approach facilitates an appreciation of how changes in the structure of S-GAG can regulate physiology as well as pathology.^(8,13)^ Mass spectrometry and gel electrophoresis procedures can further be used to establish the length of an oligosaccharide chain and the presence of specific functional groups.^(1,2,16)^ Sample preparation for these sensitive techniques often requires enzymatic treatments to produce oligosaccharide sequences for subsequent analysis.^(7,17-20)^

There are two techniques for ionizing large biological molecules: first, matrix-assisted desorption laser ionization, a mass spectrometry technique based on desorption and laser ionization of proteins aided by a matrix, which analyzes the mass by the flight time of ion analysis of the tube (time of flight – ToF), and second, the mass spectrometry based on ionization by electric pulses in liquid medium (electrospray ionization mass spectrometry – ESI-MS).^(1,2,21)^

## OBJECTIVE

To describe an experimental method of analyzing sulfated glycosaminoglycans in neoplastic and non-neoplastic tissue specimens from patients with colorectal carcinoma, using electrospray ionization mass spectrometry-based method analysis. While there are various techniques for studying the interaction of sulfated glycosaminoglycans with proteins, the broad applicability of this method offers an insight into how changes in cell surface and extracellular sulfated glycosaminoglycans composition and sequence influence the ability of the cells and tissues to dynamically alter responses to signaling molecules in colorectal cancer tissues.

## METHODS

Tissue samples were obtained from 64 adult patients with colorectal carcinoma without metastatic disease, who had been consecutively operated on during the period of 2005 to 2006, in the Department of surgery of our organization. Thirty-seven (57.8%) patients were female. The mean age was 68.5±7.3 years (range: 44 to 81 years). All the patients were caucasian. The carcinoma was located in the rectum in 32 (50.0%) patients, and in the colon, in the other 32 (50.0%) − 17 (26.6%) in the right colon and 15 (23.4%) in the left colon.

For mass spectrometry assays, we used two tissue samples of the large intestine of each patient: one tissue sample obtained from a representative macroscopic area of the colorectal carcinoma, and another from non-neoplastic mucosa, located 10cm cranially to the neoplasia.

The neoplastic and non-neoplastic tissues, biopsies from patients, were ground and homogenized with twice the volume of acetone, for 24 hours, and acetone was changed four times. These homogenates were kept at room temperature for 48 hours. The material was then centrifuged at 1,300Xg, for 20 minutes, discarding the supernatant and drying the precipitate under vacuum. The dry powder tissue obtained was weighed and submitted to proteolysis in the presence of maxatase (Biocon, Rio de Janeiro, (RJ), Brazil), 2mg/100mg of dry tissue, in a 0.05M Tris HCl buffer, pH 8.0, containing 0.15M NaCl, at a ratio of 10mL enzyme solution per 1g of dry tissue. Trichloroacetic acid TCA (10% end concentration) was added to the mixture and maintained at 4°C, for 15 minutes. After incubation for approximately 48 hours, at 60°C, nucleic acids and proteins remaining in solution were precipitated in an ice bath by adding TCA (10% end concentration). After 10 minutes, the solution was centrifuged (1,300Xg, during 15 minutes) to remove the precipitate. Two volumes of methanol for precipitation of S-GAG were added slowly and with constant stirring to the supernatant. The supernatant containing S-GAG was obtained after centrifugation (10 minutes, 3,500Xg, 4°C). S-GAG were precipitated by adding two volumes of methanol (24 hours, -20°C). The precipitate was collected by centrifugation (20 minutes, 3,500Xg, 4°C), dried, and dissolved in distillated water (50mL of water per gram of acetone powder). An aliquot of this solution was separated, dried under vacuum, and resuspended in DNase solution to inactivate the nucleic acids.

### Identification and quantification of sulfated glycosaminoglycans

Aliquots of S-GAG (50 to 100µL) were incubated with condroitinases B, AC, and heparitinases I, II, and III, isolated from *Flavobacterium heparinum*,^(18)^ in 0.05M ethylenediamine-acetate buffer (EDA), pH 8.0, with a final volume of 30µL. After 16 hours incubation at 3rC, the volume was divided into two parts: one aliquot (5µL) was subjected to electrophoresis on agarose gel, and the other was used for identification of compounds and their fragments by mass spectrometry.

Both extracellular matrix and cellular S-GAG were co-purified following the described procedure. S-GAG were identified and quantified by agarose gel electrophoresis in 0.05M 1.3-diaminopropane-acetate buffer (PDA), pH 9.0. After electrophoresis, S-GAG were precipitated in agarose gel using 0.1% acetyl-trimethylammonium-bromide (Sigma-Aldrich, Saint Louis, Missouri, United States), for 2 hours, at room temperature. The gel was dried and stained with toluidine blue (0.1% in acetic acid: ethanol: water; 0.1:5:4.9, v/v). S-GAG quantification was carried out by densitometry at 530nm. The extinction coefficients of the S-GAG were calculated using standards of chondroitin 4-sulfate (from whale cartilage), DS (from pig skin), and HS (from bovine pancreas) (Seikagaku Kogyo Co., Tokyo, Japan). The agarose gel electrophoresis method error was of the order of 5%. Identification of the S-GAG was based on migration of the compounds compared with that of the standards. The identity of the different S-GAG was confirmed by degradation with specific lyases: chondroitinases B, AC (Seikagaku Kogyo Co., Tokyo, Japan) and heparatinases obtained as previously described.^(19)^

The substrate specificity of chondroitinases B, AC were CS A and C, chondroitin, and hyaluronic acid. The chondroitinase AC acts in the regions of CS, while chondroitinase B acts in the regions of DS containing links 13(1-4) between N-acetylgalactosamine and QL-iduronic acid.^(18)^

Heparitinases I and II degrade HS, and unsaturated disaccharides formed as degraded products are a non-sulfate compound (ΔDiHS-OS) and a small amount of uronic acid-glucosamine-N-sulfate (ΔDiHS-NS). Heparitinase I acts on N-acetylated or N-sulfated glucosamine-glucuronic acid links of the HS. Sulfate groups at the 6-position of the glucosamine moiety of the HS chains seem to be impeditive for heparitinase I action. Heparitinase II acts upon HS producing disulfated, N-sulfated, and N-acetylated-6-sulfated disaccharides, and small amounts of N-acetylated disaccharide. Heparitinase II acts preferentially upon N-6-sulfated glucosamine-glucuronic acid links. Heparitinase III degrades HS, and unsaturated disaccharides formed as degraded products are the non-sulfate compound (ΔDiHS-OS) and uronic acid-glucosamine-N-sulfate (ΔDiHS-NS). The total degradation of HS is only achieved by the combined action of these heparitinases.^(17)^

The produced disaccharides were purified and digested with either chondroitinase B, AC or a mix of heparitinases I, II, and III, as described elsewhere.^(22)^

### Electrospray ionization mass spectrometry

The studies of ESI-MS were conducted at the Brazilian Synchrotron Light Laboratory (Campinas, São Paulo, (SP), Brazil) using the Micromass Q-TOF instrument (Micromass Inc., Milford, Massachusetts, United States) for ESI-MS analysis to detect the presence of S-GAG in the colorectal tissue samples. The composition of the electrospray solvent was acetonitrile-phosphoric acid, the same one used for S-GAG oligosaccharides.

S-GAG obtained from the tissues were degraded or not with specific lyases, diluted in acetonitrile phosphoric acid (1:1), centrifuged at 1.400Xg, injected into the ESI-MS apparatus with a mass-load ratio of 4kDa, and subjected to nebulization. The device was programmed to act in a negative way, *i.e.*, selecting only negative ions with energy in the cone of 80V and subjected to a 5µL/minute flow.

The composition of the electrospray solvent was the same used for S-GAG oligosaccharides, *i.e.*, acetonitrile-phosphoric acid, and the corresponding flow rate was 5µL/minute.

S-GAG or their degradation products were analyzed by electrophoresis on agarose gel in 0.05M PDA, pH 9.0 buffer. After electrophoresis (5V/cm, for 1 hour), the blade was dipped into a 0.1% cetrimide solution for precipitation of S-GAG, for a minimum of 2 hours.

The samples were applied to agarose gel blades, and were then subjected to electrophoresis (5V/cm for hour) in the box cooled to 5°C. Since these compounds possess anionic charge, the origin of the gel corresponded to the negative pole.

The gel was dried under a hot air stream and artificial lighting, and after approximately 90 minutes, the compounds were stained with a toluidine blue solution, and the excess removed with a solution of 1% acetic acid and 50% ethanol.

The system of electrophoresis in agarose gel allows the identification of S-GAG, separating them according to the interaction with the cap. The 0.05M PDA buffer, pH 9.0, distinguishes the compounds according to the differential interaction with the diamine, discriminating, in order of decreasing electrophoretic mobility: CS, DS, and HS.^(10)^ Quantification of S-GAG was performed by densitometry using a wavelength of 560nm.

This study was conducted in accordance with the regulations of the Human Research Ethics Committees under number 090/04 at our organizations and with the ethical principles of the Declaration of Helsinki of 1975, as revised in 2000.

### Statistical analysis

The values obtained in the study of each continuous variable were organized and expressed as mean and standard deviation. Absolute and relative frequencies were used for categories. Statistical associations were determined using Student’s *t*-test. The statistical significance level was set at 5% (p<0.05), and the data were analyzed using the Statistical Package for Social Sciences software (SPSS™, Chicago, IL, United States), version 15.0.

## RESULTS

S-GAG obtained from tissues of colorectal carcinoma and non-neoplastic tissue were identified and quantified by agarose gel electrophoresis, after extraction and precipitation by proteolysis. Thus, this assay avoided any material losses that normally occur during the process.

**Figure 1:**
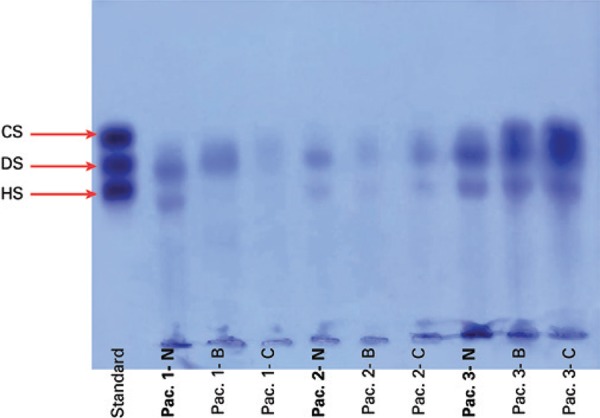
Sulphated glycosaminoglycan electrophoresis of colorectal tissues (5µg each)

CS: chondroitin sulphate; DS: dermatan sulphate; HS: heparan sulphate. Standard, non-neoplastic colorectal tissue (N), colorectal carcinoma tissue (B, C).

In the agarose gel, the S-GAG extracted from colorectal tissue showed three electrophoretic bands. Two of them corresponded to the migration of HS and DS, and the third band was delayed when compared to the standard CS migration, making it difficult to separately quantify the compounds CS and DS. Consequently, the two galactosaminoglycans (CS + DS) were quantified together as a single band (Figure 1). However, to ensure the presence of DS and CS in the colorectal tissue samples, the S-GAG extracted from the degradation by enzymatic proteolysis with maxatase were subjected to incubation with specific enzymes (lyases), and the degradation products obtained were analyzed by ESI-MS.

**Figure 2:**
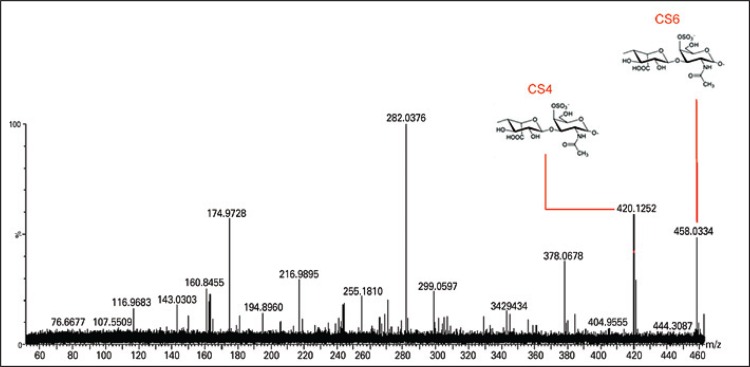
Mass spectroscopy to identify chondroitin sulphate oligosaccharides. Chondroitin sulphate oligosaccharides and disaccharides were obtained after chondroitinase AC digestion. The products were compared with the standard of chondroitin-4-sulphated and chondroitin-6-sulphated products

**Figure 3:**
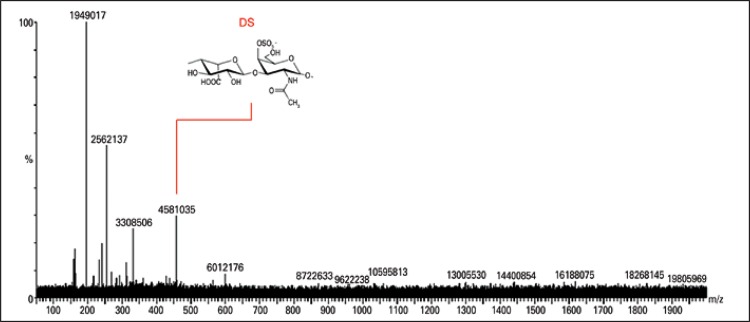
Mass spectroscopy to identify dermatan sulphate oligosaccharides. Dermatan sulphate oligosaccharides and disaccharides were obtained after chondroitinase B digestion. The products were compared with the standard of dermatan sulphate products

**Figure 4:**
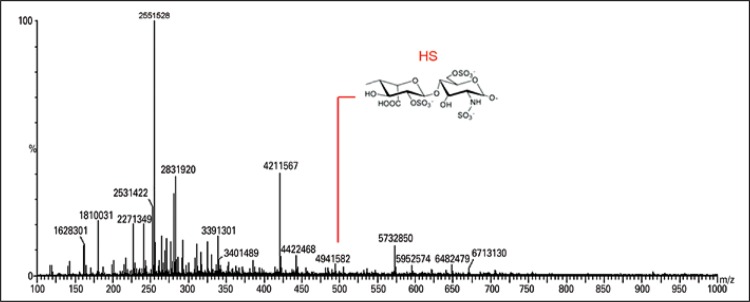
Mass spectroscopy to identify heparan sulphate oligosaccharides. Heparan sulphate oligosaccharides and disaccharides were obtained after heparitinase I and II digestion. The products were compared with the standard of heparan sulphate products

The ESI-MS was obtained concerning the degradation of colorectal tissue samples with chondroitinase AC and confirmed the presence of CS disaccharides and their possible fragments (Figure 2). The lyases produced Δ4,5-unsaturated HexAβ1-3 GalNAc-sulfate. The ion at m/z 378 corresponded to the loss of SO3 from m/z 458. Other ions were labeled, the their presence indicated that the electrospray desolvation conditions were too energetic and thus resulted in dissociation of the CS/DS disaccharides. Likewise, the degradation of colorectal tissue with chondroitinase B showed the presence of DS disaccharides and possible fragments (Figure 3). The degradation of colorectal tissue samples with the enzyme heparitinase I, II, and III showed the presence of HS, as well as possible oligosaccharide fragments characteristic of disaccharides and HS (Figure 4). Heparitinases from *Flavobacterium heparinum* degraded a linkage region containing L-iduronic acid, D-glucosamine-N-sulfated, and 6-sulfated.

CS: chondroitin sulphate.

DS: dermatan sulphate.

HS: heparan sulphate.

The ESI-MS showed a characteristic disaccharide profile obtained from CS, DS, and HS, indicating the presence of S-GAG in the colorectal tissues analyzed. However, some peaks in the ESI-MS were not characterized as sugar fragments, indicating the presence of protein fragments obtained from PG proteolysis.

**Figure 5:**
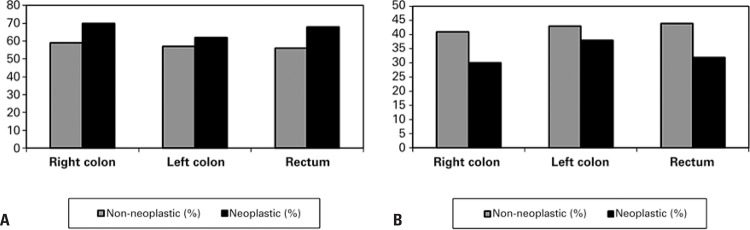
Sulphated glycosaminoglycan profile in neoplastic and non-neoplastic tissue specimens from colorectal carcinoma. (A) Galactosaminoglycans (chondroitin sulphate and dermatan sulphate) increased in neoplastic tissues when compared to non-neoplastic tissues (p=0.01). (B) Heparan sulphate decreased in neoplastic tissues when compared to non-neoplastic tissue (p=0.03)

The percentages of S-GAG as galactosaminoglycans (CS and DS) and HS in neoplastic and non-neoplastic colorectal tissue specimens, obtained by agarose gel electrophoresis and degradation with specific lyases (chondroitinases B, AC and heparatinases), in the different locations of colorectal carcinoma, are showed in table 1 and figure 5.

**Table 1:** Sulphated glycosaminoglycan* profile in neoplastic and non-neoplastic tissue specimens from colorectal carcinoma

Glycosaminoglycans**	Right colon		Left colon		Rectum	
		
	Neoplastic (%)	Non-neoplastic (%)	Neoplastic (%)	Non-neoplastic (%)	Neoplastic (%)	Non-neoplastic (%)
Galactosaminoglycans	70	59	62	57	68	56
Heparan sulphate	30	41	38	43	32	44
Total	100	100	100	100	100	100

* The values of sulphated glycosaminoglycans in neoplastic and non-neoplastic tissue specimens from patients with colorectal carcinoma are expressed as percentage of total sulphated glycosaminoglycans (%); ** the quantification was performed by agarose gel electrophoresis, as described in Methods, and the values represent triplicates obtained from two different assays.

The results showed that the average amount of CS and DS increased in the neoplastic tissue in the right colon, left colon, and rectum compared to the normal tissue in these segments of the large bowel (p=0.01). On the other hand, the average amount of HS decreased in the neoplastic tissue in the right colon, left colon, and rectum, compared to the normal tissue in these segments of the large bowel (p=0.03).

## DISCUSSION

Interactions between glycans and proteins are crucial to many biological regulatory processes.^(4,5,23)^ The development of analytical methodologies that enable structural characterization of S-GAG oligosaccharides fostered improved understanding of the specificity of these biomolecular interactions and biological functions.^(3-5,16)^

S-GAG are difficult molecules for analysis by mass spectrometric due to their high diversity and complexity in saccharide composition, polydispersity, and sequence heterogeneity.^(11,23,24)^

Various procedures were devised to tag glycans selectively at the reducing end with a group that would enhance the sensitivity of detection and facilitate chromatographic separations.^(6,9,25,26)^ These methods provide new opportunities for the development of glycomic approaches to study the structure and function of the HS, CS, and DS family of glycans.^(1-3,10,14)^ Thus, structural characterization of oligosaccharides from PG and other glycoproteins is greatly enhanced through the use of mass spectrometric and gel electrophoresis.^(1,2,14)^ Sample preparations for these sensitive techniques often require enzymatic treatments to produce oligosaccharide sequences for subsequent analysis.^(2,3,17)^Overall, the modularity of these techniques provides ease and flexibility for use in conjunction with mass spectrometric and electrophoretic tools for glycomic research studies.^(10,12)^

This study describes a technique to elucidate the structural information inherent in S-GAG species. First, the use of depolymerizing enzymes that cleave polysaccharides at specific sites is described. Then, the agarose gel electrophoresis technique was employed to characterize the S-GAG species present in an enzymatically cleaved polysaccharide sample. Mass spectrometric procedures were further used to establish the length of an oligosaccharide chain and the presence of specific functional oligosaccharide and disaccharide groups.

To our knowledge, the present study was the first to describe the application of ESI-MS in the identification of S-GAG from the extracellular matrix of non-neoplastic and neoplastic colorectal tissues. After digestion with chondroitinase AC and chondroitinase B, the pool of disaccharides could be directly separated by liquid chromatography onto a porous graphitized carbon column and identified by online electrospray mass spectrometric under negative ionization conditions. The relative intensities of the fragment ions obtained by the mass spectrometric-based method allow one to distinguish the sulphate position. Calibration with standard disaccharides allows quantification of the different isomers.

During tumor growth, transformed cells remodel the extracellular space for dynamic growth conditions.^(5,8)^As PG and S-GAG are negatively charged ubiquitous components of all matrices, and during the process of tumor growth, they constitute a real interface in the dynamic exchange between transformed cells and normal tissue.^(4,5,8,9)^

Actually, PG and S-GAG have contradictory properties in tumor progression during stromal reaction.^(15,25)^Stromal PG have antiproliferative properties, while the S-GAG liberated after PG degradation, and its degradation oligosaccharides promote cancer cell migration.^(2,17)^ The results of this study showed that neoplastic tissues present higher amounts of CS/DS, as compared to the non-neoplastic tissues, while HS was decreased in neoplastic tissues. This finding of reduced amount of HS corroborates with literature data demonstrating HS as important glycosaminoglycan for cell-cell recognition and cell cycle inhibition.^(5,27)^ On the other hand, increased CS and DS in neoplastic tissues may be explained by increased cell proliferation, due to the fact that blocking chondroitin synthesis results in cytokinesis defects in early embryogenesis.^(4,5,8)^ Reversion of cytokinesis is often observed in chondroitin-depleted embryos, and cell division eventually stops, resulting in early embryonic death, proving that CS is required for embryonic cytokinesis and cell division.^(4,5,21,28)^ The progression of the tumor may change S-GAG and PG, especially depending on the initial expression pattern of the source cell, which leads to the loss or alteration of the expression pattern.^(21,28)^ This occurs because most PG present a hybrid molecule, and thereby, the core of the protein can be either glycanated with CS or HS, an event that changes the function of the molecule.^(4,5)^ Furthermore, protein core fragments, emerging from PG degradation, may also possess biological activity.^(6,29)^

The broad applicability of this powerful platform offers an insight into how changes in cell surface, extracellular matrix, and S-GAG composition and sequence influence the ability of the cells and tissues to dynamically alter responses to signaling molecules in colorectal carcinoma.^(5,8,15)^ Thus, this approach provides a window into how changes at the structural disaccharides composition level of each glycosaminoglycan could affect the cellular phenotype in the malignant disease or non-neoplastic tissue, due to the fact that glycosaminoglycan chain composition defines specific binding and promotes defined biological function.^(6,15,16,30)^

The technique of ESI-MS allowed the study of structural fragments generated by ionizing the sample, and the agarose gel electrophoresis was able to identify and quantify S-GAG (CS, DS, and HS) in colorectal carcinoma and colorectal non-neoplastic tissues.^(24)^ This method provides new opportunities for developing glycomic approaches to study the structure and function of CS, DS, and HS in colorectal carcinoma and colorectal non-neoplastic tissues.^(4,8,21)^

## CONCLUSION

The structural profile of glycosaminoglycans in the colorectal tissue can be determined by electrospray ionization mass spectrometry after proteolysis and electrophoresis. The increased amount of chondroitin and dermatan suggests that they play a role in neoplastic spread, while the reduced amount of heparan indicate its role as an inhibitor of neoplastic proliferation.

Considering the critical role of sulphated glycosaminoglycans (S-GAG) in tumor proliferation and metastasis, the knowledge of their biological behavior could help to develop a therapeutic target for colorectal cancers to retard tumor progression by modulating the deregulated biosynthetic and catabolic pathways of sulphated glycosaminoglycan chains through novel chemical biology approaches.
