# Supplementary material for: Glycomics expression analysis of sulfated glycosaminoglycans of human colorectal cancer tissues and non-neoplastic mucosa by electrospray ionization mass spectrometry
Source: Einstein (Sao Paulo). 2015 Oct-Dec;13(4):510–7. doi: 10.1590/S1679-45082015AO3477 (PMC4878623; doi:10.1590/S1679-45082015AO3477)
Supplement: Translation

# Translation: Análise da expressão de glicosaminoglicanos sulfatados no tecido humano neoplásico colorretal e na mucosa não neoplásica por espectrometria de massa por ionização por *electrospray*

**Authors:** Ana Paula Cleto Marolla, Jaques Waisberg, Gabriela Tognini Saba, Daniel Reis Waisberg, Fernando Beani Margeotto, Maria Aparecida da Silva Pinhal

**Affiliations:** 1 Universidade Federal de São Paulo, São Paulo, SP, Brasil.; 2 Hospital do Servidor Público Estadual, São Paulo, SP, Brasil.; 3 Faculdade de Medicina do ABC, Santo André, SP, Brasil.; 4 Faculdade de Medicina da Universidade de São Paulo, São Paulo, SP, Brasil.

**Keywords:** Glicosaminoglicanas, Proteoglicanas, Condroitina, Dermatan sulfato, Heparitina sulfato, Matriz extracelular, Neoplasias colorretais, Espectrometria de massas por ionização por *electrospray*

## Abstract

**Objetivo:**

Determinar a presença de glicosaminoglicanos na matriz extracelular do tecido conjuntivo colorretal neoplásico e não neoplásico, tendo em vista seu papel central no desenvolvimento e na progressão dos tumores.

**Métodos:**

Amostras de tecidos colorretais neoplásicos e não neoplásicos foram obtidas de 64 pacientes operados com carcinoma colorretal sem metástases a distância. As expressões de heparan sulfato, sulfato de condroitina e sulfato de dermatan e seus fragmentos foram analisadas por espectrometria de massa por ionização por *electrospray*, com técnica de extração e quantificação de glicosaminoglicanos após proteólise e eletroforese. Para análise estatística, utilizaram-se média, desvio padrão e teste *t* de Student.

**Resultados:**

Em gel de agarose, os glicosaminoglicanos extraídos de tecido colorretal mostraram três bandas eletroforéticas. A espectrometria de massa por ionização por *electrospray* mostrou fragmentos de dissacarídeos característicos de glicosaminoglicanos e indicou sua característica estrutural. Alguns picos na espectrometria de massa por ionização por *electrospray* não foram caracterizados como fragmentos de açúcares, sugerindo a presença de fragmentos de proteínas estruturais dos proteoglicanos, formadas durante a purificação dos glicosaminoglicanos. A quantidade média de condroitina e dermatan aumentou no tecido neoplástico em relação ao tecido normal (p=0,01). Por outro lado, a quantidade média de heparan foi menor no tecido neoplásico em relação ao tecido normal (p=0,03).

**Conclusão:**

O método empregado permitiu determinar o perfil estrutural dos glicosaminoglicanos nas amostras. Tecidos neoplásicos apresentaram maiores quantidades de sulfato de condroitina e sulfato de dermatan em comparação com os não neoplásicos, enquanto o sulfato de heparan foi encontrado em menores quantidades nos tecidos neoplásicos.

## INTRODUÇÃO

A homeostase da matriz extracelular depende da integridade e do arranjo de colágenos, glicoproteínas, proteoglicanos (PG) e glicosaminoglicanos sulfatados (S-GAG).^(1-3)^ Os S-GAG se ligam a proteínas específicas e alteram seus papéis essenciais em desenvolvimento, organogênese, crescimento celular, adesão celular, inflamação, modulação da angiogênese, apoptose, controle do ciclo celular e sinalização dos fatores de crescimento.^(4-7)^Ademais, os S-GAG desempenham um papel vital em cada passo da progressão tumoral, permitindo que células cancerosas proliferem, escapem da resposta imunológica, invadam tecidos vizinhos e formem metástases em locais distantes do sítio primário.^(5,8,9)^ O sulfato de condroitina (SC) e sulfato de dermatan (SD), ambos galactosaminoglicanos, e o sulfato de heparan (SH), um glicosaminoglicano, são os S-GAG envolvidos nesses eventos biológicos.^(10-12)^

Várias técnicas foram revisadas para a identificação seletiva de glicanos na extremidade redutora, com um grupo que poderia aumentar a sensibilidade de detecção e facilitar as separações cromatográficas.^(3,13,14)^ No entanto, a análise de S-GAG e PG derivados das pequenas quantidades de tecidos ou células, e o desenvolvimento de métodos que caracterizem esses carboidratos têm progredido de forma mais lenta.^(1-3)^

Espectrometria de massa é uma técnica analítica usada para identificar compostos desconhecidos, quantificar materiais conhecidos e elucidar as propriedades químicas e estruturais de moléculas, com base na geração de íons detectados posteriormente.^(3,15)^Essa abordagem permite avaliar como as mudanças na estrutura de S-GAG podem regular a fisiologia e a patologia.^(8,13)^ Os procedimentos de espectrometria de massa e eletroforese em gel podem também ser usados para estabelecer o comprimento da cadeia de um oligossacarídeo e a presença de grupos funcionais específicos.^(1,2,16)^O preparo de amostras para essas técnicas sensíveis requer frequentemente tratamentos enzimáticos para produzir sequências de oligossacarídeos para análises posteriores.^(7,17-20)^

Há duas técnicas para a ionização de grandes moléculas biológicas: primeiro, a ionização/dessorção a laser assistida por matriz, uma técnica de espectrometria de massa baseada na dessorção e ionização a laser de proteínas, com a ajuda de uma matriz, que analisa a massa por meio do tempo de voo (ToF - *time of flight*) de íons do tubo; e segundo, a ionização baseada em espectrometria de massa por pulsos elétricos, em meio líquido (espectrometria de massa com ionização por *electrospray* – ESI-EM).^(1,2,21)^

## OBJETIVO

Descrever um método experimental de análise de glicosaminoglicanos sulfatados em espécimes de tecidos neoplásicos e não neoplásicos de pacientes com carcinoma colorretal, usando análise baseada no método de espectrometria de massa por ionização por *electrospray*. Embora existam várias técnicas para o estudo da interação de glicosaminoglicanos sulfatados com proteínas, a ampla aplicabilidade desse método oferece uma perspectiva da maneira em que alterações na superfície da célula e na composição e sequência de glicosaminoglicanos sulfatados extracelulares influenciam na capacidade das células e tecidos de mudar dinamicamente suas respostas a moléculas de sinalização em tecidos de câncer colorretal.

## MÉTODOS

Foram obtidas amostras de tecidos de 64 pacientes adultos com carcinoma colorretal sem doença metastática, operados consecutivamente durante o período de 2005 a 2006, no departamento de cirurgia da nossa instituição. Trinta e sete (57,8%) pacientes eram mulheres. A média de idade foi 68,5±7,3 anos (variação de 44 a 81 anos). Todos os pacientes eram brancos. O carcinoma estava localizado no reto em 32 (50,0%) pacientes e no cólon nos outros 32 (50,0%) − 17 (26,6%) no cólon direito e 15 (23,4%) no cólon esquerdo.

Para os ensaios de espectrometria de massa, usamos duas amostras de tecido do intestino grosso de cada paciente: uma amostra de tecido obtida de uma área macroscópica, representativa do carcinoma colorretal, e outra da mucosa não neoplásica, a 10cm em direção ao crânio, em relação à neoplasia.

Os tecidos de biópsias dos pacientes foram triturados e homogeneizados com o dobro do volume de acetona, por 24 horas, e a acetona foi trocada quatro vezes. Esses preparados homogeneizados foram mantidos em temperatura ambiente por 48 horas. O material foi, então, centrifugado a 1.300Xg, por 20 minutos; o sobrenadante, descartado; e secado o precipitado com vácuo. O tecido em pó seco assim obtido foi pesado e submetido à proteólise na presença de maxatase (Biocon, Rio de Janeiro, (RJ), Brasil) 2mg/100mg de tecido seco, em um solução tampão de 0,05M Tris HCl, pH 8,0, contendo 0,15M NaCl, na proporção de 10mL da solução enzimática por 1g de tecido seco. Foi acrescentado ácido tricloroacético TCA (10% concentração final) à mistura, sendo mantido a 4°C, por 15 minutos. Após a incubação por aproximadamente 48 horas, a 60°C, os ácidos nucleicos e proteínas remanescentes na solução foram precipitados em um banho de gelo pela adição de TCA (10% concentração final). Após 10 minutos, a solução foi centrifugada (1.300Xg por 15 minutos) para remover o precipitado. Dois volumes de metanol para precipitação de S-GAG foram adicionados vagarosamente ao sobrenadante, misturando sem interrupção. O sobrenadante contendo S-GAG foi obtido após centrifugação (10 minutos, 3.500Xg, 4°C). Os S-GAG foram precipitados pela adição de dois volumes de metanol (24 horas, -20°C). O precipitado foi coletado por centrifugação (20 minutos, 3.500Xg, 4°C), secado e dissolvido em água destilada (50mL de água por grama de pó de acetona). Uma alíquota dessa solução foi separada, secada a vácuo e novamente suspensa em solução de DNase para inativar os ácidos nucleicos.

### Identificação e quantificação dos glicosaminoglicanos sulfatados

As alíquotas de S-GAG (50 a 100µL) foram incubadas com condroitinases B, AC e heparitinases I, II e III, isoladas de *Flavobacterium heparinum*,^(18)^ em 0,05M de tampão de etileno diamina-acetato (EDA), pH 8,0, com volume final de 30µL. Após 16 horas de incubação a 3rC, o volume foi dividido em duas partes: uma alíquota (5µL) foi submetida à eletroforese em agarose gel, e a outra foi usada para identificação dos compostos e seus fragmentos por espectrometria de massa.

Os S-GAG celulares e da matriz extracelular foram copurificados após o procedimento descrito. Os S-GAG foram identificados e quantificados por eletroforese em agarose gel em tampão de 1,3-diamino propano-acetato (PDA) 0,05M, pH 9,0. Após eletroforese, os S-GAG foram precipitados em agarose gel usando 0,1% de brometo de acetil trimetil amônia (Sigma-Aldrich, Saint Louis, Missouri, Estados Unidos), por 2 horas, em temperatura ambiente. O gel foi secado e corado com azul de toluidina (0,1% em ácido acético: etanol: água; 0,1:5:4,9, v/v). A quantificação de S-GAG foi feita por densitometria a 530nm. Os coeficientes de extinção dos S-GAG foram calculados usando padrões de 4-sulfato de condroitina (de cartilagem de baelia), SD (de pele de porco) e SH (de pâncreas bovino) (Seikagaku Kogyo Co., Tóquio, Japão). O erro do método de eletroforese em gel de agarose foi da ordem de 5%. A identificação dos S-GAG foi baseada na migração dos compostos em comparação à dos padrões. A identidade dos diferentes S-GAG foi confirmada pela degradação com liases específicas: condroitinases B, AC (Seikagaku Kogyo Co., Tokyo, Japão) e heparatinases obtidas conforme descrição anterior.^(19)^

A especificidade de substrato de condroitinases B, AC foram sulfatos de condroitina A e C, condroitina e ácido hialurônico. A condroitinase AC age nas regiões de SC, enquanto a condroitinase B age nas regiões de SD, que contêm ligações 13(1-4) entre N-acetilgalactosamina e ácido QL-idurônico.^(18)^

As heparitinases I e II degradam SH, e dissacarídeos insaturados formados como produtos de degradação são um composto não sulfatado (ΔDiHS-OS) e uma pequena quantidade de ácido urônico-glucosamina-N-sulfato (ΔDiHS-NS). A heparitinase I age sobre as ligações entre ácidos N-acetilados ou N-sulfatados de glucosamina-ácido glucurônico do SH. Os grupos sulfato na posição 6 da porção de glucosamina das cadeias de SH parecem ser impeditivos para a ação de heparitinase I. A heparitinase II age sobre SH produzindo dissacarídeos dissulfatados, N-sulfatados e N-acetilados-6-sulfatados, além de pequenas quantidades de dissacarídeos N-acetilados. A heparitinase II age preferencialmente sobre as ligações entre ácidos N-6-sulfatados de glucosamina-ácido glucurônico. Já a heparitinase III degrada SH, e os dissacarídeos insaturados formados como produtos degradados são o composto não sulfatado (ΔDiHS-OS) e ácido urônico-glucosamina-N-sulfato (ΔDiHS-NS). A degradação total de SH somente é conseguida pela ação combinada destas heparitinases.^(17)^

Os dissacarídeos produzidos foram purificados e digeridos com condroitinase B, AC ou uma mistura de heparitinases I, II e III, conforme já publicação.^(22)^

### Espectrometria de massa por ionização por *electrospray*

Os estudos de ESI-EM foram conduzidos no Laboratório Nacional de Luz Síncrotron (Campinas, São Paulo, (SP), Brasil) usando o instrumento Micromass Q-TOF (Micromass Inc., Milford, Massachusetts, Estados Unidos) para análises de ESI-EM a fim de detectar a presença de S-GAG nas amostras de tecido colorretal. A composição do solvente do *electrospray *foi acetonitrila-ácido fosfórico − a mesma usada para os oligossacarídeos S-GAG.

Os S-GAG obtidos dos tecidos foram degradados ou não com liases específicas, diluídas em acetonitrila/ácido fosfórico (1:1), centrifugados a 1.400Xg, injetados no aparelho de ESI-EM com razão massa-carga de 4kDa e submetidos à nebulização. O dispositivo foi programado para agir de forma negativa, ou seja, selecionava apenas íons negativos com energia no cone de 80V e submetidos a um fluxo de 5µL/minuto.

A composição do solvente do *electrospray* foi a mesma usada para os oligossacarídeos S-GAG, ou seja, acetonitrila-ácido fosfórico, e a taxa de fluxo correspondente foi de 5µL/minuto.

Os S-GAG ou seus produtos de degradação foram analisados por eletroforese em agarose gel em 0,05M de solução tampão de PDA, pH 9,0. Após eletroforese (5V/cm, por 1 hora), a lâmina foi mergulhada em uma solução de cetrimida 0,1% para precipitação de S-GAG, por um período mínimo de 2 horas.

As amostras foram aplicadas a lâminas de agarose gel e submetidas à eletroforese (5V/cm por 1 hora) na caixa resfriada até 5°C. Como esses compostos possuem carga aniônica, a origem do gel correspondia ao polo negativo.

O gel foi secado sob um fluxo de ar quente e luz artificial e, após aproximadamente 90 minutos, os compostos foram tingidos com uma solução de azul de toluidina, sendo excesso removido com uma solução de ácido acético 1% e etanol 50%.

O sistema de eletroforese em agarose gel permite a identificação dos S-GAG, separando-os segundo a interação com o *cap*. O tampão de PDA 0,05M, pH 9,0, distingue os compostos segundo a interação diferencial com a diamina, discriminando, em ordem decrescente de motilidade eletroforética: SC, SD e SH.^(10)^ A quantificação dos S-GAG foi feita por densitometria usando um comprimento de ondas de 560nm.

Este estudo foi conduzido de acordo com os regulamentos dos Comitês de Ética em Pesquisa Humana número 090/04 nas nossas instituições e com os princípios éticos da Declaração de Helsinque de 1975, conforme revisão de 2000.

### Análise estatística

Os valores obtidos no estudo de cada variável foram organizados e expressos na forma de média e desvio padrão. Frequências absoluta e relativa foram usadas para as categorias. As associações estatísticas foram determinadas usando o teste *t* de Student. O nível de significância estatística foi determinado em 5% (p<0,05), e os dados foram analisados usando *software Statistical Package for Social Sciences* - SPSS™, (Chicago, Illinois, Estados Unidos), versão 15.0.

## RESULTADOS

Os S-GAG obtidos de tecidos de carcinoma colorretal e tecidos não neoplásicos foram identificados e quantificados por eletroforese em agarose gel, após extração e precipitação por proteólise. Assim, este ensaio evitou quaisquer perdas materiais que normalmente ocorrem durante o processo.

No gel de agarose, os S-GAG extraídos do tecido colorretal mostraram três bandas eletroforéticas. Duas delas corresponderam à migração de SH e SD, e a terceira banda foi mais lenta quando comparada ao padrão SC de migração, tornando difícil a quantificação separada dos compostos SC e SD. Consequentemente, os dois galactosaminoglicanos (SC + SD) foram quantificados juntos de uma banda única (Figura 1). No entanto, para garantir a presença de SD e SC nas amostras de tecido colorretal, os S-GAG extraídos da degradação por proteólise enzimática com maxatase foram submetidos à incubação com enzimas específicas (liases), e os produtos de degradação obtidos foram analisados por ESI-EM.

**Figura 1:** Eletroforese de glicosaminoglicano sulfatado de tecidos colorretais (5µg cada)

SC: sulfato de condroitina; SD: sulfato de dermatan; SH: sulfato de heparan. Padrão, tecido colorretal não neoplásico (N), tecido colorretal com carcinoma (B, C).

O ESI-EM foi obtido com relação à degradação de tecidos de amostras colorretais com condroitinase AC e confirmou a presença de dissacarídeos SC e seus possíveis fragmentos (Figura 2). As liases produziram sulfato Δ 4,5-insaturado HexAβ1-3 GalNAc. O íon em m/z 378 correspondeu à perda de SO3 de m/z 458. Outros íons foram rotulados, e a presença deles indicou que as condições de dessolvatação do *electrospray* foram energéticas demais e resultaram na dissociação dos dissacarídeos SC/SD. Da mesma forma, a degradação de tecido colorretal com condroitinase B mostrou a presença de dissacarídeos SD e possíveis fragmentos (Figura 3). A degradação de amostras de tecido colorretal com a enzima heparitinase I, II e III mostrou a presença de SH, possíveis fragmentos de oligossacarídeos característicos dos dissacarídeos e SH (Figura 4). As heparitinases de *Flavobacterium heparinum* degradaram a região de ligação contendo ácido L-idurônico, D-glucosamina-N-sulfatado e 6-sulfatado.

**Figura 2:** Espectroscopia de massa para identificar oligossacarídeos de sulfato de condroitina. Os oligossacarídeos e dissacarídeos de sulfato de condroitina foram obtidos após digestão com condroitinase AC. Os produtos foram comparados com o padrão de produtos de condroitina-4-sulfatada e condroitina-6-sulfatada

SC: sulfato de condroitina.

**Figura 3:** Espectroscopia de massa para identificar oligossacarídeos de sulfato de dermatan. Os oligossacarídeos e dissacarídeos de sulfato de dermatan foram obtidos após digestão com condroitinase B. Os produtos foram comparados com o padrão de produtos de sulfato de dermatan

SD: sulfato de dermatan.

**Figura 4:** Espectroscopia de massa para identificar oligossacarídeos de sulfato de heparan. Oligossacarídeos e dissacarídeos de sulfato de heparan após digestão com heparitinase I e II. Os produtos foram comparados com o padrão de produtos de sulfato de heparan

SH: sulfato de heparan.

O ESI-EM mostrou um perfil característico de dissacarídeo obtido de SC, SD e SH, indicando a presença de S-GAG nos tecidos colorretais analisados. Porém alguns picos no ESI-EM não foram caracterizados como fragmentos de açúcar, indicando a presença de fragmentos de proteína obtidos da proteólise de PG.

As porcentagens de S-GAG, na forma de galactosaminoglicanos (SC e SD) e SH em espécimes de tecido colorretal neoplásicos e não neoplásicos, obtidos por eletroforese em agarose gel e degradação com liases específicas (condroitinases B, AC e heparatinases), nos diferentes locais de carcinoma colorretal, são mostradas na tabela 1 e figura 5.

**Tabela 1:** Perfil de glicosaminoglicano* sulfatado em espécimes de tecidos neoplásicos e não neoplásicos de carcinoma colorretal

Glicosaminoglicanos**	Cólon direito		Cólon esquerdo		Reto	
		
	Neoplástico (%)	Não neoplásico (%)	Neoplástico (%)	Não neoplásico (%)	Neoplástico (%)	Não neoplásico (%)
Galactosaminoglicanos	70	59	62	57	68	56
Sulfato de heparan	30	41	38	43	32	44
Total	100	100	100	100	100	100

* Os valores de glicosaminoglicanos sulfatados em espécimes de tecidos neoplásicos e não neoplásicos de pacientes com carcinoma colorretal são expressos na forma de porcentagem do total de glicosaminoglicanos sulfatados (%); **a quantificação foi feita por eletroforese em agarose gel, conforme descrito na seção métodos, e os valores representam triplicatas obtidas de dois diferentes ensaios.

**Figura 5:** Perfil de glicosaminoglicanos sulfatados em espécimes de tecidos neoplásicos e não neoplásicos de carcinoma colorretal. (A) Galactosaminoglicanos (sulfato de condroitina e sulfato de dermatan) aumentados em tecidos neoplásicos quando comparados a tecidos não neoplásicos (p=0,01). (B) Sulfato de heparan diminuído em tecidos neoplásicos quando comparados a tecidos não neoplásicos

Os resultados mostraram que a quantidade média de SC e SD aumentou no tecido neoplásico do cólon direito, cólon esquerdo e reto, em comparação ao tecido normal nesses segmentos do intestino grosso (p=0,01). Por outro lado, a quantidade média de SH diminuiu no tecido neoplásico no cólon direito, cólon esquerdo e reto, quando comparados ao tecido normal nesses segmentos do intestino grosso (p=0,03).

## DISCUSSÃO

As interações entre glicanos e proteínas são cruciais para muitos processos biológicos regulatórios.^(4,5,23)^ O desenvolvimento de metodologias analíticas, que possibilitam a caracterização estrutural de oligossacarídeos S-GAG, levou a um melhor entendimento sobre a especificidade dessas interações biomoleculares e funções biológicas.^(3-5,16)^

Os S-GAG são moléculas difíceis de analisar por espectrometria de massa, devido à sua alta diversidade e à complexidade na composição de sacarídeos, polidispersidade e heterogeneidade de sequência.^(11,23,24)^

Diversos procedimentos foram criados para rotular os glicanos seletivamente na extremidade redutora, com um grupo que realçaria a sensibilidade de detecção e facilitaria separações cromatográficas.^(6,9,25,26)^ Esses métodos fornecem novas oportunidades para o desenvolvimento de abordagens dos glicosaminoglicanos para estudar a estrutura e a função da família de SH, SC e SD de glicanos.^(1-3,10,14)^ Assim, a caracterização estrutural de oligossacarídeos de PG e outras glicoproteínas é bem maior com espectrometria de massa e eletroforese em gel.^(1,2,14)^ As amostras preparadas para essas técnicas sensíveis requerem frequentemente tratamentos enzimáticos para produzir sequências de oligossacarídeos para a próxima análise.^(2,3,17)^No geral, a modularidade dessas técnicas facilita e flexibiliza o uso, junto de ferramentas de espectrometria de massa e eletroforese para pesquisas com glicosaminoglicanos.^(10,12)^

Este estudo descreve uma técnica para elucidar a informação estrutural inerente nas espécies de S-GAG. Primeiro, discorre sobre o uso de enzimas de despolimerização que causam clivagem de polissacarídeos em pontos específicos. Depois, a técnica de eletroforese em agarose gel foi empregada para caracterizar as espécies de S-GAG presentes em uma amostra de polissacarídeo clivada por meio de enzimas. Os procedimentos de espectrometria de massa foram também usados para estabelecer o comprimento da cadeia de um oligossacarídeo e a presença de grupos específicos de oligossacarídeos e dissacarídeos funcionais.

Até onde sabemos, este foi o primeiro estudo a descrever a aplicação de ESI-EM na identificação de S-GAG da matriz extracelular de tecidos colorretais não neoplásicos e neoplásicos. Após digestão com condroitinase AC e condroitinase B, o *pool *de dissacarídeos pôde ser separado diretamente por cromatografia líquida em uma coluna de grafite porosa e identificado *on-line* por espectrometria de massa por* electrospray*, em condições de ionização negativas. As intensidades relativas dos íons dos fragmentos obtidos pelo método baseado em espectrometria de massa permitem a distinção da posição do sulfato. A calibração com dissacarídeos padrão permite a quantificação dos diferentes isômeros.

Durante o crescimento do tumor, as células transformadas remodelam o espaço extracelular para haver condições de crescimento dinâmico.^(5,8)^Os PG e S-GAG são componentes ubíquos de carga negativa em todas as matrizes e, durante o processo de crescimento tumoral, constituem uma verdadeira interface para a troca dinâmica entre células transformadas e tecido normal.^(4,5,8,9)^

De fato, PG e S-GAG têm propriedades contraditórias na progressão tumoral durante a reação estromal.^(15,25)^ Os PG estromais têm propriedades antiproliferativas, enquanto os S-GAG liberados após a degradação de PG e os oligossacarídeos de sua própria degradação promovem migração celular.^(2,17)^ Os resultados deste estudo mostraram que tecidos neoplásicos apresentam maiores quantidades de SC/SD em comparação com os tecidos não neoplásicos, enquanto SH estava diminuído em tecidos neoplásicos. Esse achado de quantidade reduzida de SH corrobora os dados da literatura e demonstra SH como importante para o reconhecimento entre células e a inibição do ciclo celular.^(5,27)^ Por outro lado, níveis aumentados de SC e SD em tecidos neoplásicos podem ser explicados pela proliferação celular aumentada, pois o bloqueio da síntese de condroitina resulta em defeitos citocinéticos na embiogênese inicial.^(4,5,8)^ A reversão da citocinese é frequentemente observada em embriões com depleção de condroitina, e a divisão celular por fim cessa, levando à morte embrionária precoce, o que comprova que SC é necessário para a citocinese e a divisão celular embrionárias.^(4,5,21,28)^ A progressão do tumor pode alterar os S-GAG e PG, especialmente na dependência do padrão inicial de expressão da célula fonte, levando à perda ou à alteração do padrão de expressão.^(21,28)^ Isso ocorre porque a maior parte dos PG apresenta molécula híbrida e, portanto, o centro da proteína pode ser glicado com SC ou SH, um evento que muda as funções da molécula.^(4,5)^ Ademais, fragmentos do centro da proteína, emergentes da degradação do PG, também podem ter atividade biológica.^(6,29)^

A ampla aplicabilidade desta poderosa plataforma oferece uma perspectiva da maneira como alterações de composição e sequência na superfície célula, matriz extracelular e S-GAG influenciam na capacidade de as células e os tecidos de mudarem dinamicamente suas respostas a moléculas de sinalização no carcinoma colorretal.^(5,8,15)^ Assim, esta abordagem fornece uma janela de visão para a forma como alterações no nível de composição estrutural de dissacarídeos de cada glicosaminoglicano poderiam afetar o fenótipo celular na doença maligna ou tecidos não neoplásicos, já que a composição da cadeia de glicosaminoglicanos define ligações específicas e promove função biológica definida.^(6,15,16,30)^

A técnica de ESI-EM permitiu o estudo de fragmentos estruturais gerados pela ionização da amostra, e a eletroforese em agarose gel foi capaz de identificar e quantificar os S-GAG (SC, SD e SH) em tecidos colorretais com carcinoma e não neoplásicos.^(24)^ Este método oferece novas oportunidades para o desenvolvimento de abordagens de glicosaminoglicanos no estudo da estrutura e função de SC, SD e SH em tecidos colorretais com carcinoma e não neoplásicos.^(4,8,21)^

## CONCLUSÃO

O perfil estrutural dos glicosaminoglicanos no tecido colorretal pode ser determinado por espectrometria de massa por ionização por *electrospray *após proteólise e eletroforese. A quantidade aumentada de condroitina e dermatan sugere que ambos desempenhem um papel na disseminação neoplásica, enquanto a quantidade reduzida de heparan indica seu papel como inibidor da proliferação neoplásica.

Considerando o papel vital dos glicosaminoglicanos sulfatados na proliferação de tumores e metástases, o conhecimento de seu comportamento biológico pode ajudar a desenvolver um alvo terapêutico para diferentes tipos de câncer colorretal e retardar a progressão tumoral pela modulação das vias biossintéticas e catabólicas das cadeias de glicosaminoglicanos sulfatados por meio de novas abordagens biológicas químicas.
